# Supplementary material for: TST conversions and systemic interferon-gamma increase after methotrexate introduction in psoriasis patients
Source: PLoS One. 2020 Dec 3;15(12):e0242098. doi: 10.1371/journal.pone.0242098 (PMC7714364; doi:10.1371/journal.pone.0242098)
Supplement: S1 Table — (DOCX) [file pone.0242098.s002.docx]

S1 Table – Measures of associations between positive and negative IGRA results and the average values of numerical variables before MTX treatment.

|  | **IGRA before** | |  |
| --- | --- | --- | --- |
| **Variable** | **Positive** | **Negative** | **P value** |
|  |  |  |  |
|  |  |  |  |
| **Age (years): mean ± SD** | 47.63 ± 20.17 | 47.12 ± 17.38 | P ^(1)^ = 0.953 |
|  |  |  |  |
| **Disease duration (years): median (P25; P75)** | 3.00 (1.00; 11.25) | 8 (20.00 3.00;) | P ^(2)^ = 0.109 |
|  |  |  |  |
| **Weight (kg): mean ± SD** | 75.85 ± 26.30 | 74.35 ± 15.60 | P ^(1)^ = 0.864 |
|  |  |  |  |
| **BMI: mean ± SD** | 27.79 ± 5.96 | 27.69 ± 4.74 | P ^(1)^ = 0.967 |
|  |  |  |  |
| **Blood glucose (mg/dl): mean ± SD** | 90.37 ± 10.70 | 104.72 ± 22.65 | P ^(2)^ = 0.095 |
|  |  |  |  |
| **CRP (mg/dl) before: median (P25; P75)** | 1.20 (0.36; 4.95) | 0.41 (0.10; 2.44) | P ^(2)^ = 0.282 |
|  |  |  |  |
| **ESR (mm) before: median (P25; P75)** | 24.00 (11.25; 30.50) | 10.00 (4.50; 16.25) | P ^(2)^ = 0.038 * |
|  |  |  |  |
| **IFN-γ (pg/ml)** **before: mean ± SD** | 15.57 ± 10.19 | 16.06 ± 7.36 | P ^(2)^ = 0.877 |
|  |  |  |  |
| **TNF-α (pg/ml) before: median (P25; P75)** | 0.00 (0.00; 2.77) | 0.00 (0.00; 3.08) | P ^(2)^ = 0.598 |
|  |  |  |  |
| **PASI before: median (P25; P75)** | 12.60 (7.05; 16.28) | 13.20 (10.40; 21.40) | P ^2)^ = 0.696 |
|  |  |  |  |
| **PASI after: median (P25; P75)** | 2.80 (1.05; 8.25) | 1.50 (0.80; 3.70) | P ^(2)^ = 0.427 |
|  |  |  |  |
| **PASI reduction: median (P25; P75)** | 8.40 (3.60; 12.68) | 10.00 (7.20; 20.00) | P ^(2)^ = 0.409 |
|  |  |  |  |
| **PASI reduction %: mean ± SD** | 67.22 ± 25.64 | 78.94 ± 19.01 | P ^(2)^ = 0.241 |
|  |  |  |  |

TST, tuberculin skin test; BMI body mass index; ESR, erythrocyte sedimentation rate; CRP, C-reactive protein; IFN-γ, interferon-gamma; TNF-α, tumour necrosis factor-alpha; PASI, psoriasis area and severity index. (*) P < 5 % (1) Student's t-test with equal variances. (2) Mann-Whitney test.
