# Supplementary material for: TST conversions and systemic interferon-gamma increase after methotrexate introduction in psoriasis patients
Source: PLoS One. 2020 Dec 3;15(12):e0242098. doi: 10.1371/journal.pone.0242098 (PMC7714364; doi:10.1371/journal.pone.0242098)
Supplement: S2 Table — (DOCX) [file pone.0242098.s003.docx]

S2 Table – Measures of associations between positive and negative TST results and the average values of numerical variables before MTX treatment

|  | **TST before** | |  |
| --- | --- | --- | --- |
| **Variable** | **Positive** | **Negative** | **P value** |
|  |  |  |  |
|  |  |  |  |
| **Age (years): mean ± SD** | 45.94 ± 17.73 | 47.66 ± 18.08 | P ^(1)^ = 0.841 |
|  |  |  |  |
| **Disease duration (years): median (P25; P75)** | 5.50 (2.50; 23.50) | 8.00 (2.00; 16.00) | P ^(2)^ = 0.915 |
|  |  |  |  |
| **Weight (kg): mean ± SD** | 81.90 ± 25.32 | 72.44 ± 15.34 | P ^(1)^ = 0.273 |
|  |  |  |  |
| **BMI: mean ± SD** | 28.22 ± 5.81 | 27.56 ± 4.78 | P ^(1)^ = 0.781 |
|  |  |  |  |
| **Blood glucose (mg/dl): mean ± SD** | 94.20 ± 8.49 | 103.51 ± 23.56 | P ^(1)^ = 0.358 |
|  |  |  |  |
| **CRP (mg/dl) before: median (P25; P75)** | 0.75 (0.15; 5.43) | 0.54 (0.11; 2.63) | P ^(2)^ = 0.879 |
|  |  |  |  |
| **ESR (mm) before: median (P25; P75)** | 13.00 (6.75; 28.50) | 12.00 (4.50; 20.75) | P ^(2)^ = 0.661 |
|  |  |  |  |
| **IFN-γ (pg/ml)** **before: mean ± SD** | 20.51 ± 9.14 | 14.50 ± 7.12 | P ^(2)^ = 0.106 |
|  |  |  |  |
| **TNF-α (pg/ml)** **before: median (P25; P75)** | 0.00 (0.00; 2.58) | 0.13 (0.00; 3.08) | P ^(2)^ = 0.275 |
|  |  |  |  |
| **PASI before: median (P25; P75)** | 14.20 (11.15; 16.28) | 11.70 (7.20; 21.40) | P ^2)^ = 0.547 |
|  |  |  |  |
| **PASI after: median (P25; P75)** | 1.60 (0.78; 6.68) | 2.10 (1.10; 3.70) P ^(2)^ = 0.357 | P ^(2)^ = 1.000 |
|  |  |  |  |
| **PASI reduction: median (P25; P75)** | 10.65 (9.73; 12.68) | 8.10 (4.50; 20.00) | P ^(2)^ = 0.357 |
|  |  |  |  |
| **PASI reduction %: mean ± SD** | 79.69 ± 17.96 | 75.00 ± 21.99 | P ^(2)^ = 0.746 |
|  |  |  |  |

TST, tuberculin skin test; BMI body mass index; ESR, erythrocyte sedimentation rate; CRP, C-reactive protein; IFN-γ, interferon-gamma; TNF-α, tumour necrosis factor-alpha; PASI, psoriasis area and severity index. (1) Student's t-test. (2) Mann-Whitney test.
