# Supplementary material for: TST conversions and systemic interferon-gamma increase after methotrexate introduction in psoriasis patients
Source: PLoS One. 2020 Dec 3;15(12):e0242098. doi: 10.1371/journal.pone.0242098 (PMC7714364; doi:10.1371/journal.pone.0242098)
Supplement: S3 Table — (DOCX) [file pone.0242098.s004.docx]

S3 Table – Measures of associations between positive and negative IGRA results and the average values of numerical variables after MTX treatment.

|  | **IGRA after** | |  |
| --- | --- | --- | --- |
| **Variable** | **Positive** | **Negative** | **P value** |
|  |  |  |  |
|  |  |  |  |
| **Age (years): mean ± SD** | 57.19 ± 19.66 | 43.38 ± 15.70 | P ^(1)^ = 0.078 |
|  |  |  |  |
| **Disease duration (years): median (P25; P75)** | 4.00 (2.00; 16.00) | 8.00 (2.75; 17.75) | P ^(2)^ = 0.734 |
|  |  |  |  |
| **Weight (kg): mean ± SD** | 64.57 ± 16.06 | 17.67 ± 78.65 | P ^(1)^ = 0.080 |
|  |  |  |  |
| **BMI: mean ± SD** | 24.66 ± 4.37 | 28.90 ± 4.71 | P ^(1)^ = 0.051 |
|  |  |  |  |
| **Blood glucose (mg/dl): mean ± SD** | 99.99 ± 20.23 | 101.78 ± 22.07 | P ^(2)^ = 0.918 |
|  |  |  |  |
| **CRP (mg/dl) before: median (P25; P75)** | 0.30 (0.14; 0.80) | 0.50 (0.12; 0.94) | P ^(2)^ = 0.832 |
|  |  |  |  |
| **ESR (mm) before: median (P25; P75)** | 10.00 (13.00 5.00;) | 7.00 (2.00; 15.50) | P ^(2)^ = 0.443 |
|  |  |  |  |
| **IFN-γ (pg/ml)** **before: mean ± SD** | 17.98 ± 3.66 | 19.15 ± 10.65 | P ^(1)^ = 0.667 |
|  |  |  |  |
| **TNF-α (pg/ml)** **before: median (P25; P75)** | 5.50 (0.00; 8.72) | 1.87 (0.33; 6.10) | P ^(2)^ = 0.576 |
|  |  |  |  |
| **PASI before: median (P25; P75)** | 13.80 (10.70; 24.90) | 12.30 (9.45; 15.53) | P ^2)^ = 0.350 |
|  |  |  |  |
| **PASI after: median (P25; P75)** | 1.20 (0.60; 3.30) | 2.10 (1.18; 5.25) | P ^(2)^ = 0.214 |
|  |  |  |  |
| **PASI reduction: median (P25; P75)** | 10.80 (10.00; 21.60) | 8.20 (4.38; 14.03) | P ^(2)^ = 0.163 |
|  |  |  |  |
| **PASI reduction %: mean ± SD** | 86.08 ± 15.27 | 72.26 ± 21.77 | P ^(2)^ = 0.137 |
|  |  |  |  |

TST, tuberculin skin test; BMI body mass index; ESR, erythrocyte sedimentation rate; CRP, C-reactive protein; IFN-γ, interferon-gamma; TNF-α, tumour necrosis factor-alpha; PASI, psoriasis area and severity index. (1) Student's t-test with equal variances. (2) Mann-Whitney test.
