# Supplementary material for: TST conversions and systemic interferon-gamma increase after methotrexate introduction in psoriasis patients
Source: PLoS One. 2020 Dec 3;15(12):e0242098. doi: 10.1371/journal.pone.0242098 (PMC7714364; doi:10.1371/journal.pone.0242098)
Supplement: S4 Table — (DOCX) [file pone.0242098.s005.docx]

S4 Table – Measures of associations between positive and negative TST results and the average values of numerical variables after MTX treatment.

|  | **TST after** | |  |
| --- | --- | --- | --- |
| **Variable** | **Positive** | **Negative** | **P value** |
|  |  |  |  |
|  |  |  |  |
| **Age (years): mean ± SD** | 46.95 ± 16.59 | 47.48 ± 19.05 | P ^(1)^ = 0.979 |
|  |  |  |  |
| **Disease duration (years): median (P25; P75)** | 4.00 (8.00 2.00;) | 13.00 (2.75; 17.75) | P ^(1)^ = 0.196 |
|  |  |  |  |
| **Weight (kg): mean ± SD** | 78.76 ± 19.15 | 71.52 ± 17.24 | P ^(2)^ = 0.331 |
|  |  |  |  |
| **BMI: mean ± SD** | 27.44 ± 4.41 | 27.93 ± 5.45 | P ^(2)^ = 0.813 |
|  |  |  |  |
| **Blood glucose (mg/dl): mean ± SD** | 96.82 ± 8.36 | 104.78 ± 27.26 | P ^(1)^ = 0.989 |
|  |  |  |  |
| **CRP (mg/dl) before: median (P25; P75)** | 0.68 ± 1.20 | 0.65 ± 0.56 | P ^(1)^ = 0.387 |
|  |  |  |  |
| **ESR (mm) before: median (P25; P75)** | 2.00 (2.00 1.00;) | 2.00 (2.00 1.75;) | P ^(1)^ = 0.448 |
|  |  |  |  |
| **IFN-γ (pg/ml)** **before: mean ± SD** | 22.91 ± 11.68 | 15.61 ± 4.99 | P ^(1)^ = 0.072 |
|  |  |  |  |
| **TNF-α (pg/ml) before: median (P25; P75)** | 6.50 (0.00; 8.12) | 1.87 (0.49; 5.6) | P ^(1)^ = 0.218 |
|  |  |  |  |
| **PASI before: median (P25; P75)** | 13.8 (10.7; 20.4) | 12.45 (7.05; 18.4) | P ^(1)^ = 0.411 |
|  |  |  |  |
| **PASI after: median (P25; P75)** | 2.00 (0.80; 5.70) | 1.80 (1.00; 4.05) | P ^(1)^ = 0.989 |
|  |  |  |  |
| **PASI reduction: median (P25; P75)** | 10.50 (8.30; 12.90) | 8.10 (4.38; 18.05) | P ^(1)^ = 0.366 |
|  |  |  |  |
| **PASI reduction %: mean ± SD** | 86.58 (60.96; 92.92) | 78.92 (52.5; 93.96) | P ^(1)^ = 0.758 |
|  |  |  |  |

TST, tuberculin skin test; BMI body mass index; ESR, erythrocyte sedimentation rate; CRP, C-reactive protein; IFN-γ, interferon-gamma; TNF-α, tumour necrosis factor-alpha; PASI, psoriasis area and severity index. (1) Mann-Whitney test. (2) Student's t-test with equal variances.
