# Supplementary figures and images for: TST conversions and systemic interferon-gamma increase after methotrexate introduction in psoriasis patients
Source: PLoS One. 2020 Dec 3;15(12):e0242098. doi: 10.1371/journal.pone.0242098 (PMC7714364; doi:10.1371/journal.pone.0242098)

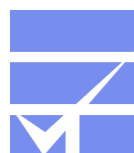

## CONSORT 2010 Flow Diagram

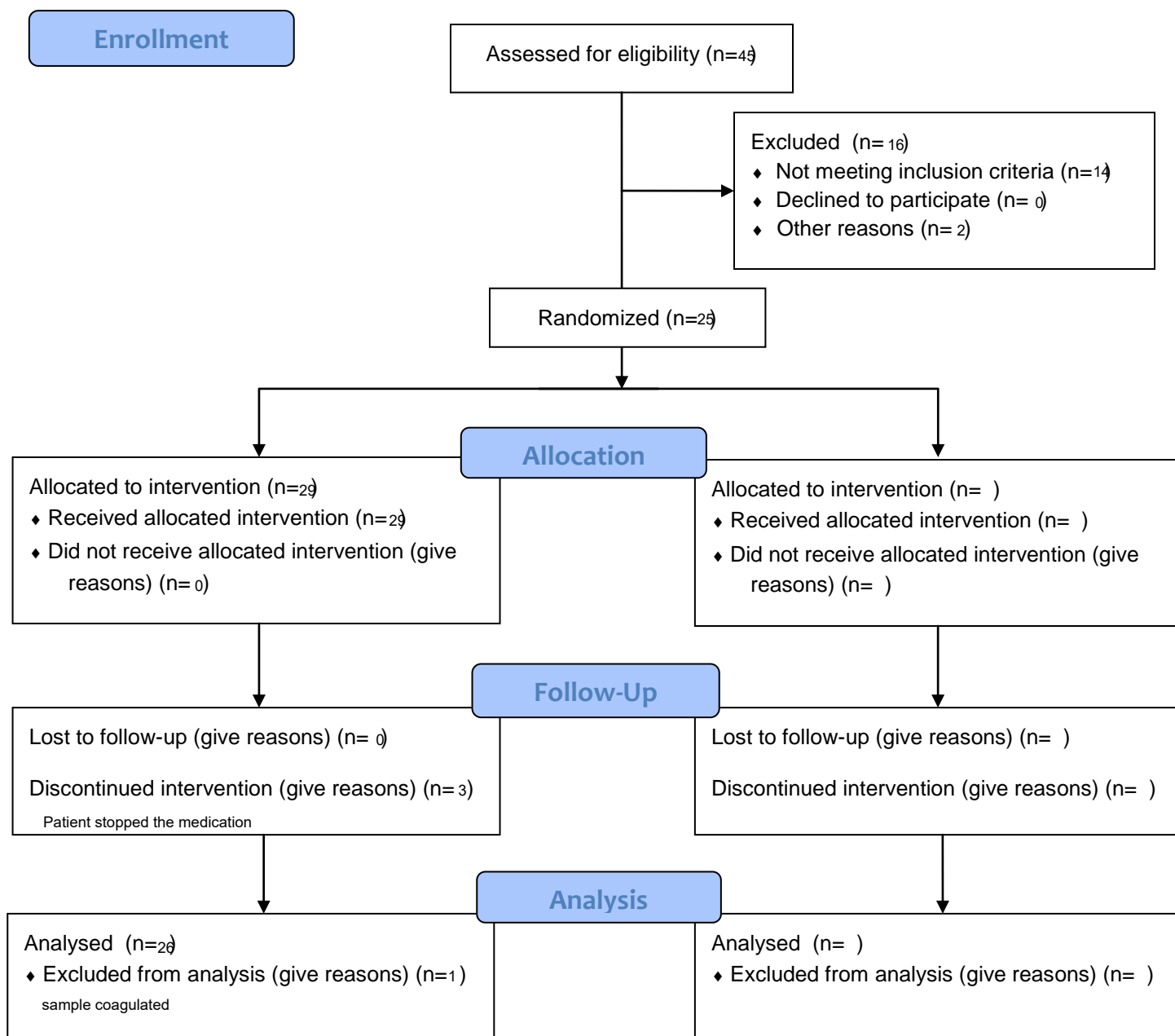

Supplement: S1 Fig — (PDF) [file pone.0242098.s006.pdf]
