# Supplementary material for: TST conversions and systemic interferon-gamma increase after methotrexate introduction in psoriasis patients
Source: PLoS One. 2020 Dec 3;15(12):e0242098. doi: 10.1371/journal.pone.0242098 (PMC7714364; doi:10.1371/journal.pone.0242098)
Supplement: S1 File — (DOCX) [file pone.0242098.s007.docx]

POSTGRADUATE DEPARTMENT OF TROPICAL MEDICINE

**FEDERAL UNIVERSITY OF PERNAMBUCO -UFPE**


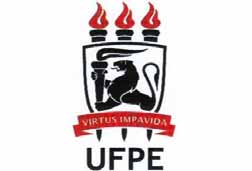


"INTERFERENCE OF METHOTREXATE ON THE QUANTIFERON TB-GOLD IN-TUBE TEST IN THE SCREENING OF LATENT TUBERCULOSIS AND ITS RELATIONSHIP TO THE CLINICAL, INFLAMMATORY AND IMMUNOLOGICAL ACTIVITY OF PSORIASIS."

P.h.D. project

Author: Vanessa Lucília Silveira de Medeiros M.D.

Advisor: Prof. Dr. Vera Magalhães da Silveira

Recife 2014

**Research Team**

| Name of the professional | *Degree and position* | *RESEARCH ACTIVITIES* |
| --- | --- | --- |
| *Dr. Vanessa Lucília Silveira de Medeiros* | - M.D. Specialist in internal medicine and dermatology - Master in Tropical Medicine - Coordinator of the Psoriasis outpatient clinic of Dermatology UFPE. - Fellow at the Henry Ford Hospital-Detroit 2010 | - Reaserch Project desing - Coordination of the parties involved - Sample selection - Clinical evaluation |
| *Dr. Vera Magalhães da Silveira* | - M.D. Infectologist - Ph.D. in Tropical Medicine Unifesp- SP - Vice PhD in Tropical Medicine -Chairman of Postgraduate department of Tropical Medicine-CCS UFPE | - Research Project advisor |
| *dR. Líbia CRISTINA Rocha vilela MOURA* | - M.D infectologist - PhD in Tropical Medicine | - Research Project co-advisor |
| *vALDÊNIA mARIA OLIVEIRA DE SOUZA* | - Graduated in biomedical sciences - Associate Prof. of Dept. of Pharmaceutical Sciences - Researcher at the Laboratory of Immunopathology Keizo Asami-LIK - Chairman of Postgraduate Department of Tropical Medicine-CCS UFPE | - Performing Elisa tests for cytokines |
| *Lílian Maria Lapa Montenegro* | - Graduated in Biology - Ph.D. in Public Health from the Oswaldo Cruz Foundation. - Expertise in Public Health. - Coordinator of PIBIC / FIOCRUZ-CPqAM. - Researcher in the laboratory of Immunoepidemiology, Department of Immunology. Aggeu Magalhães Research Center (CPqAM) -Fiocruz | - Performing the Quantiferon-Tb gold test |
| *Reginaldo Gonçalves de lima neto* | - Graduated in Biology - Postdoctoral degree in microbiology - Specialist in clinical analysis - Permanent member of the Graduate Program in Biology, Pathology, and Biotechnology. - Adjunct Professor, Department of Tropical Medicine, Federal University of Pernambuco. | - Application and reading of the tuberculin test. - Collection of blood for IGRA and cytokines |

**Summary**

| ***1*** | ***Rationale & background information*** | | | | **1** |
| --- | --- | --- | --- | --- | --- |
|  |  | | | |  |
| ***2*** | ***Study goals and objectives*** | | | | *3* |
|  | Main objective | | | | *3* |
|  | Specific objectives | | | | *3* |
|  | Secondary objectives | | | | *4* |
|  |  | | | |  |
| ***3*** | ***Study design*** | | | | **5** |
|  | 3.1 | Study period | | | 5 |
|  | 3.2 | Sampling type and sample size definition | | | 6 |
|  | 3.3 | Eligibility criteria | | | 7 |
|  |  |  | | |  |
| ***4*** | ***Methods of Collection*** | | | | ***8*** |
|  | 4.1 | Collection protocol | | | 8 |
|  | 4.2 | Definition of the variables | | | 12 |
|  | 4.3 | Laboratory Techniques | | | 15 |
|  |  | 4.3.1 PASI | | | 15 |
|  |  | 4.3.2 | | Cytokines | 15 |
|  |  | 4.3.3 | | QuantiFERON TB Gold In-Tube test | 16 |
|  |  | 4.3.4 | | Tuberculin Skin Test | 17 |
|  |  |  | |  |  |
| ***5*** | ***Data analyses plan*** | | | | **15** |
|  |  | | | |  |
| ***6*** | ***Budge*** | | | | 17 |
|  |  | | | |  |
| ***7*** | ***References*** | | | | **18** |
|  |  | | | |  |
| ***8*** | ***Appendices*** | | | | **25** |
|  | Appendix 1 | | Patient free and informed consent form | | *25* |
|  | Appendix 2 | | Clinical Data Record | | *27* |
|  | Appendix 3 | | Questionnaire for TB screening | | 29 |
|  | Appendix 4 | | Laboratory Follow-up Record | | 30 |
|  | Appendix 5 | | PASI Score Calculation | | 31 |
|  | Appendix 6 | | Laboratory Data Record | | 32 |
|  | Appendix 7 | | Hospital Consent to carry out the study | | 33 |
|  | Appendix 8 | | Term of data confidentiality | | 34 |
|  | Appendix 9 | | Authorization to access Patient Data | | 35 |
|  |  | | | |  |

#### Rationale & background information

The use of tumor necrosis factor-alpha (TNF-α) blockers for the treatment of patients with psoriasis increases the risk of developing tuberculosis (TB) in 25-fold compared to those who do not receive these medications (SOLOVIC, 2013). Therefore, current consensuses advocate screening for latent tuberculosis infection (TBLI) with a tuberculin skin test (TST) before initiating the use of TNF-α blocking drugs (DAUDEN et al., 2016; CONSENSO…, 2012; COMISSÃO..., 2009; GRUPO..., 2002)

However, the application of TST in patients with psoriasis presents many problems. On one hand, there are reports of tests with false-negative results, being questioned whether these would be due to immunosuppression, energy, or technical errors. In these cases, patients were allowed to use TNF-α blockers and may develop Latent Tuberculosis infection (LTBI) (FORTALEZA et al, 2008; GOUJON et al., 2010; SHAIKHA et al., 2012; ABREU et al., 2013; YOO et al., 2014). On the other hand, TT has a higher frequency of positive and strong reactors in this population than controls, to increase the number of patients unnecessarily submitted to TB chemoprophylaxis (TISIURI et al., 2009; BASSUKAS et al. 2011).

Until today, the causal factors responsible for increasing the frequency of positive TST results observed in patients with psoriasis have not been defined. One of the hypotheses suggests the interference of the immunological activation of the Th17 and Th22 pathways by the disease (LIMA et al., 2011; KARATAS et al., 2016). However, there are no studies that have evaluated the relationship between TT outcome and levels of cytokines involved in pathways common to psoriasis and tuberculosis.

It has been suggested that the interferon-γ (IGRA) release assay should replace the use of TST in the TBLI screening because it has greater sensitivity and specificity (BELO et al., 2014). However, the studies comparing patients with psoriasis and normal controls observed a lower concordance of TST(LAFFITTE et al, 2009; LIMA, 2013). It was then questioned whether the reason was the interference of the disease itself (LAFFITTE et al., 2009; DE ANDRADE LIMA et al., 2011), immunosuppression (BARTALEZZI et al, 2009; RAMOS et al., 2013, CONSTANTINO et al., 2013, HELWIG et al, 2012; CHIU, HSUEH, TSAI, 2011) or epidemiological factors such as age (TAVAST et al., 2012), contact with cattle ((RAMOS *et* *al*, 2013; LAFFITTE *et* *al*, 2009)) or vaccination with the Bacilli of Calmette-Guérin (SAUZULLO *et al*., 2013; LAFFITTE *et* *al*, 2009).

The test possibly affected by immunosuppression is also controversial. Some studies have found that immunosuppression affects the results of IGRA (BERTALEZZI et al, 2009, RAMOS et al, 2013, CONSTANTINO et al, 2013, HELWIG et al, 2012) while others the TS T (TAVAST et al., 2012) and there are still those that did not find an influence on both tests (MARTYN-SIMMONS et al., 2012; LATORRE et al., 2014). As many patients with psoriasis are screened for TBLI using an immunosuppressant, more often methotrexate (MTX) (DAUDÉN et al., 2016), it becomes important to elucidate whether immunosuppression interferes with TBLI screening tests.

There are several reasons why the issue has not been elucidated. Most studies evaluating the effect of immunosuppression are retrospective and present factors that may influence test results (CONSTANTINO et al 2013, RAMOS et al, 2013) as individuals with varied diseases, heterogeneous BCG vaccination status and geographic region of origin. (BARTALEZZI et al, 2009, TAVAST et al, 2012, RAMOS et al, 2013).

The search for an association between the results of screening tests with factors that could infer the impact of immunosuppression such as the degree of clinical control, variation in the inflammatory and immunological markers received little attention so far. Sauzullo et al. (2013) described in-vitro a reduction in the levels of interferon-gamma (IFN-γ) specific for TB inversely proportional to levels of a tumor necrosis factor alpha blocking drug.

Regarding the inflammatory activity, Constantino et al. (2013) found an association between undetermined IGRA and high levels of C-reactive protein (CRP) in rheumatoid arthritis, with no similar evaluation for psoriasis. The relationship of the tests with the clinical activity of the disease measured by the variation in the PASI (psoriasis area and severity index) score has not been evaluated to date.

Therefore, this study aims to compare the results of the IGRA and TST tests before and after immunosuppression with methotrexate in patients with psoriasis, with the same BCG vaccination profile, from the same geographic region, and assessing whether there is an association of these results with the clinical and inflammatory activity of the disease.

The results of the study will help to better clarify the limitations and possible biases of tuberculosis screening tests in patients with psoriasis who live in places with a high likelihood of contact with tuberculosis.

#### Study goals and objectives

- 1. **Main objectives**

Verify-in patients with psoriasis, living in an endemic area for tuberculosis and vaccinated with BCG, whether MTX significantly changes the frequency of TST and/or IGRA results and concordance between tests and whether positive tests are associated with age, disease duration in years, blood glucose, body mass index (BMI), PASI (psoriasis area and severity index), CRP, erythrocyte sedimentation rate (ESR), TNF-α, IFN-γ, IL-12, IL17 and IL 23 before and after the use of methotrexate for 12 weeks and PASI reduction after treatment.

- 1. **Specifics objectives**

Determining before and after 12 weeks of methotrexate use in psoriasis patients:

- The value of PASI
- The PASI reduction percentage
- Frequency of QuantiFERON negative, positive, indeterminate, and the test agreement.
- The TST results in mm and the test agreement.
- The agreement between TST and QuantiFERON
- The values ​​and means of the inflammatory tests (ESR and CRP)
- The levels of cytokines; TNF-α, IFN-γ, IL-12, IL17, and IL 23.

Verify if after treatment with methotrexate if there is:

- Statistical difference in frequency of positive/negative/ indeterminates TST
- Statistical difference in frequency of positive/negative/indeterminate IGRA
- Statistical difference in frequency in the agreement between TST and IGRA
- Statistical difference in PASI, cytokines and inflammatory tests levels
- Association between positive and negative tests before and after treatment with sex, age, disease duration, weight, blood glucose, BMI, ESR, CRP, PASI, TNF-α, IFN-γ, IL-12, IL17 and IL 23, absolute PASI and Percentage reduction of PASI before and after treatment.
  1. **Secondary objectives**
- Characterize the clinic and demographic characteristics of the sample following the variables sex, age, weight, BMI, disease duration in years, diabetes, associated psoriasis forms: geographical tongue, palmoplantar psoriasis, psoriasis of the scalp, inverted psoriasis, nail psoriasis, place of residence (urban or rural), contact with cattle, alcohol intake, smoking, prior contact with a tuberculosis carrier, chest X-ray abnormality suggestive of TBLI,
- Verify the association between the sustainer status with sex, age categorized, disease duration, weight, BMI, alcohol consumption, smoking, presence of other forms of psoriasis associated, blood glucose (normal, diabetic), serum levels of INF-γ, TNF-α, interleukin-12, interleukin-17, interleukin-23, ESR, CPR. The association with ESR, CPR, and cytokines will be verified before and after treatment. The variables presenting p < 0.05 will be considered a positive association.

#### Study Design

This will be a prospective, single-arm, self-controlled, quasi-experimental study associated with a cohort.

Follow-up

#### 3.1 Study period

| *Phase/year* | ***2014*** | ***2015*** | ***2016*** | ***2017*** | ***2018*** |
| --- | --- | --- | --- | --- | --- |
| Scientific project | X |  |  |  |  |
| Ethics committee submission |  | X |  |  |  |
| Data collection and follow up* |  | X** | X | X | X |
| Laboratory analysis |  |  |  | X |  |
| Data analysis |  |  |  | X | X |
| Manufacture of scientific articles |  |  |  |  | X |

* The deadline for the final analysis of data from the follow-up period will be February 2018.

* * The Collection can only be initiated after approval by the ethics committee.

3.2 Sampling type and sample size definition

The sample will be systematic random.

It will be included sequentially patients over 18 years of age with moderate to severe plaque psoriasis with the indication of methotrexate referred by dermatologists to initiate the follow-up in the outpatient clinics of the Hospital of the Federal University of Pernambuco who meet the eligibility criteria and sign the consent form.

The sample size calculation was performed considering the effect of MTX on PASI. The calculation used the formula for comparing paired differences.

To achieve a power of 80 % and a level of significance of 5 % (two-sided) for detecting an effect size of 0,65 between pair was necessary 22 pairs.

For the other variables, the objective was to verify if this effect produced on the PASI would be sufficient to produce a change in them.

#### 3.3 Eligibility criteria

***Inclusion criteria*** :

Patients over 18 years of age with moderate to severe plaque psoriasis with the indication of methotrexate referred by dermatologists to initiate the follow-up in the outpatient clinics of the Hospital of the Federal University of Pernambuco.

***Exclusion criteria:***

Absence of BCG vaccination scar, pregnant women, women breastfeeding, previous use of systemic treatment, previous use of immunosuppressants, use of topical medications for psoriasis in the last 6 months, use of drugs aggravating of psoriasis, personal history, signs or symptoms of tuberculosis, chest X-ray compatible with lung disease other than latent tuberculosis, previous malignancy, positive serology for HIV, hepatitis B or C, and liver enzyme elevation, renal clearance less than 60 ml/min, lymphopenia below 1500 lymphocytes, platelet count below 100,000, or leukopenia below 3500 leukocytes.

#### Methods of collection

#### Collection protocol

**Evaluation visit**

Patients who meet the inclusion criteria will be invited to participate in the research. The patient will only initiate the research protocol after signing the “Free and Informed Consent Term” (Appendix 1). After signing the consent form, the main researcher will collect the demographic data by interview and identification document. The data will include the date of birth, current age, comorbidities, and current medications in use (Appendix 2).

Next, the presence of the BCG vaccine scar will be checked. In the absence of the scar, the patient will be excluded. In the presence of BCG vaccine scar, other exclusion criteria will be verified; current or less than six months use of topical treatment for psoriasis and current or previous systemic treatment for psoriasis (Appendix 2). A standardized questionnaire will be used to assess the presence of active TB (Appendix 3) In the case of a positive response to any active TB symptom, history of TB treatment, or history of TB prophylaxis the patient will be excluded.

After the interview, the patients will be scheduled for collection of exams in the laboratory of the hospital of the UFPE clinics tree days before the visit one. These will include complete blood count, liver enzymes, renal function, serology of hepatitis B, C, HIV, erythrocyte sedimentation rate, quantitative C- reactive protein, rheumatoid factor, and pregnancy test for women. The chest X-ray will be performed in the radiology department of the hospital.

**Visit one**

The first visit will be done following the following protocol:

1. The main research will check the laboratory results of tests and the chest *x*-ray report. The results will be noted on the Clinical Data Record (Appendix 2) and in Laboratory follow-up Record (Appendix 4). In the case of contraindications to MTX or pulmonary radiological changes not due to possible LTBI, the patient will be excluded. The result of fasting blood glucose will be noted in mg/dl (Appendix 4).
2. The physical examination will be performed by the main research to check the severity and associated forms of psoriasis. It will be verified the presence of geographic tongue, nail psoriasis, inverted psoriasis, palm-plantar psoriasis, and scalp psoriasis. The severity of psoriasis will be quantified by the PASI score (Appendix 5)
3. The laboratory tests will be realized by *Reginaldo Gonçalves de Lima Neto*. The blood will be obtained by direct venipuncture in appropriate tubes. For the QuantiFERON-TB Gold test, one milliliter of blood will be collected in each of three tubes separately according to the sequence in the manufacturer's instructions (Cellestis, Melbourne, Australia). For the IFN-γ and TNF-α tests, 3 ml of blood will be collected in a tube with citrate. The blood samples will be identified with the inscription “Sample 1”, date, hour, and research number.
4. The blood samples will be immediately forwarded to the respective laboratories by the main research in a Styrofoam box. First, the samples for cytokines will be sent to the Laboratory of Immunopathology Keizo Asami-LIKA under the responsibility of Valdênia Souza. Second, the IGRA will be sent to the laboratory of Immunoepidemiology, Department of Immunology, Aggeu Magalhães Research Center (CPqAM) -Fiocruz under the responsibility of Lílian Maria Lapa Montenegro.
5. After the collection of blood, TST will be performed by *Reginaldo Gonçalves de Lima Neto* using the Mantoux technique described above.

**TST reading visit one**

1. The patient will be scheduled to read the result of the TST in 72 hours. The TST reading will be performed by *Reginaldo Gonçalves de Lima Neto*. The results will be photographed for archiving and noted in the laboratory data record. (Appendix 6).
2. The main research will provide the MTX prescription. The patients will be instructed to take 3 cp of 2.5 mg of MTX twice in a single day of the week, for a total dose of 15 mg per week without dose progression. Patients should start the medication the next day to avoid errors. They will be instructed to intake 5 mg of folic acid once a week in the day after the use of MTX.
3. Patients will reevaluated 30 days after starting medication to assess adverse effects. In case of adverse effects, they should attend the consultation before the deadline. Patients who discontinued the medication or skipped doses for any reason before the second visit were excluded.
4. The patient will be scheduled with one month and with tree months, before visit two, to verify the adherence to methotrexate and perform blood count, liver enzymes, renal function, and pregnancy test for women. The CPR and ESR levels will be reassessed only in three months. In case of a change in the exams or if the patient stops MTX, they will be excluded.

**|Visit two**

1. The main research will check the laboratory results of tests. The results will be noted in the clinical evaluation and laboratory records. In the case of laboratory abnormalities that contraindicate the MTX, the patient will be excluded. The result of fasting blood glucose will be reassessed to verify if it meets the criteria for diabetes.
2. The physical examination will be performed to reassess the severity of psoriasis by the PASI score with the methodology described previously.
3. The blood collection and TST application will be performed by *Reginaldo Gonçalves de Lima Neto* using the Mantoux technique. The samples will be identified with the inscription “Sample 2”, date, hour, and research number.
4. The sample will be delivered to forwarded to the respective laboratories immediately after collection, will be centrifuged, and incubated in a maximum of 1 hour. The samples from visits 1 and 2 will be stored in different trays after freezing.

**TST reading visit two**

1. The patient will be scheduled to read the result of the TST in 72 hours. The TST reading will be performed by *Reginaldo Gonçalves de Lima Neto* as the same as visit 1.

**Follow up period**

1. The patient will be scheduled every three months to check the PASI and the laboratory results: Complete blood count, liver enzymes, renal function, CPR, ESR, and pregnancy test for women. Fast blood glycemia and cholesterol will be requested according to the need of each patient.
2. The follow-up period of the study will end in February 2018. However, the patients will be kept on follow-up at the outpatient clinic for an indeterminate period, unless they abandon follow-up.
3. In case of loss of PASI response to below 50%, patients will have their medication modified according to each case by main research.

4.2 Variables definition

| Variable | Definition | Categorization |
| --- | --- | --- |
| ***TB screening*** | |  |
| **Quantiferon TB- gold first categorization** | Tests classified as positive by the manufacturer | 1. Positive |
|  | Tests classified as positive by the manufacturer | 1. Indeterminate |
|  | Tests classified as positive by the manufacturer | 1. Negative |
| **QuantiFERON TB-gold second categorization** | Tests classified as positive or undetermined | 1. Positive |
|  | Tests classified as negative | 1. Negative |
| **TST first categorization** | <5 mm | 1. Negative |
|  | 5 mm ≤ TST < 10 mm | 1. Indeterminate |
|  | ≥ 10 mm | 1. Positive |
| **TST second categorization** | <5 mm | 1. Negative |
|  | ≥ 5 mm | 1. Positive |

| *Cytokines* | |  |
| --- | --- | --- |
| **TNF-α** | in pg/ml | Average |
| **IFN-γ** | in pg/ml | Average |
| **IL -12** | in pg/ml | Average |
| **IL- 17** | in pg/ml | Average |
| **IL-23** | in pg/ml | Average |
| ***Inflammatory markers*** | |  |
| **CRP** | in mg/l | 1. ≤ 1 2. > 1 |
|  |  | Average |
| **ESR** | in mm/h | 1. ≤10 2. >10 |
|  |  | Average |
| ***Clinic*** |  |  |
| **PASI absolute** | psoriasis severity index calculated as described in methods | Mean/median |
| **PASI reduction** | Percentage reduction of PASI after the use of methotrexate | 1. <50% 2. 51-74% 3. > 75 %   Média/mediana |

| Variable | Definition | Method | Categorization |
| --- | --- | --- | --- |
| Sex | Biological gender of the patient | Interview | 1. Male 2. Female |
| Age | The time between the date of birth and study interview day, in full years | Identification document | 1. *18 ≥ x* < *30* 2. *30 ≥ x < 60* 3. *≥ 60*   Average |
| Disease duration | Estimated time between the age of onset of illness and the age at study interview day in full years | Interview | 1. < 2 year 2. 2 *≥ x <* 10 years 3. *≥ 10*  years   Average |
| alcohol intake | Have consumed more than one dose per day if the patient is a woman or 2 doses if is a man in the last 30 days. | Interview | 1. Yes 2. No |
| Smoking | I have smoked for more than one day in the last 30 days. | Interview | 1. Yes 2. No |
| Contact with Tb carrier | Characterized by a family history of TB (parents, uncles, grandparents, partners, children or own patient), know someone with TB as a neighbor, friends, co-workers or works in a high-risk TB environment | standardized questionnaire | 1. Yes 2. No |
| Place of residence | Urbanization of the place of residence | Interview | 1. Urban zone 2. Rural area |
| Contact with cattle | A person who has worked or had contact for a long time with cattle | Interview | 1. Yes 2. No |
| Nail psoriasis | Presence of pits, onycholysis, splinter hemorrhages, and hyperkeratosis not attributed to another cause in at least one nail. | Physical exam | 1. Yes 2. No |
| Geographical language | Smooth red spots on the tongue | Physical exam | 1. Yes 2. No |
| Psoriasis Scalp | Presence of psoriasis plaques on the scalp | Physical exam | 1. Yes 2. No |
| Palmoplantar psoriasis | Presence of psoriasis plaques on the palms or plants may transgress to the back | Physical exam | 1. Yes 2. No |
| Inverted psoriasis | Presence of psoriasis plaques fold areas | Physical exam | 1. Yes 2. No |
| Enthesitis | Patient presents pain on palpation of entheses without signs of arthritis | Physical exam | 1. Yes 2. No |
| Diabetes | Use of medication for diabetes or two fasting blood glucose levels above 126 mg/dl or an aleatory blood glucose level above 200 mg/dl. | Interview Laboratory tests | 1. Yes 2. No |
| Blood glucose | in mg/ml. | Laboratory tests | average |
| Chest X-ray suggestive of LTBI | Presence of retraction, calcification, hilar or mediastinal lymph node enlargement, or pleural alterations. | Image examination | 1. Yes 2. No |

| Variable | Definition | Method | Categorization |
| --- | --- | --- | --- |
| Treatment maintenance | Defined as patients who achieved 50% or more reduction in PASI score after 12 weeks of treatment and maintained this reduction until the end of the follow-up period. | Physical exam | Sustainer |
|  | Defined as patients who did not achieve a 50% reduction in PASI or those who did but lost response over the follow-up period and need a change in medication. | Physical exam | Non-sustainer |

- 1. Collection techniques
     1. **PASI**

The PASI will be carried out according to the methodology described below:

1) Each body area (head, trunk, upper limbs and lower limbs) will have their percentage of involvement determined by the researcher and will receive a number according the percentage of committed area: 1 < 10%; 2 = 10-29%; 3 = 30-49%; 4 = 50-69%; 5 = 70-89% and 6 = 90-100%

2) Each characteristic of the psoriasis plates (erythema, infiltration and flaking) will be classified at: 0 = absent, 1 = mild, 2 = moderate, and 3 = severe. The values assigned to the characteristics will be summed up for that area.

3) The value obtained in 1 will be multiplied by the value obtained in 2 for each area.

4) The product of this equation will be multiplied by a constant that depicts the proportionality of the area of each body follow-up. The head = 0.1; The trunk = 0.3; The upper limbs = 0.2 and the lower members = 0.4.

5) After the following calculation, the values will be summed up, resulting in an absolute number reflects the severity of psoriasis.

**4.3.2 Cytokines**

Three ml of blood will be collected using a vacutainer-type needle in a tube with citrate anticoagulant. The blood samples will be identified with the inscription "Sample 1" or "SAMPLE 2", date, hour, and research number. Samples will be immediately forwarded to the laboratory.

After 30 minutes of collection, the sample will be centrifuged for 15 minutes at the rate of approximately 1000 × g (or 3000 rpm) at 2-8 degrees. The serum was then separated and frozen between minus 20 ° C and minus 80 ° C. Samples will be stored in separate trays marked with the names “SAMPLE 1”, “SAMPLE 2”. The serum not used in this study will be saved for further research.

Serum levels of the cytokines will be determined quantitatively by the ELISA technique after the end of the inclusion phase. Before starting the ELISA process, calibration tests were performed. Each cytokine will be quantified twice for each sample after the calibration. The sensitivity limit of the methods is equal to 4 pg/ml for IFN-γ, 3.0 pg/ml for TNF-α and IL-23, 2 pg/ml for IL-12 and IL-17.

**4.3.3 QuantiFERON TB Gold In-Tube test**

The QuantiFERON TB Gold In-Tube test (QFT-GIT) (Cellestis, Melbourne, Vic. Australia) will be performed according to the standards described by the manufacturer

1. Collection of material: Blood samples will be obtained before the execution of TT using a vacutainer-type needle in specific collection tubes. One milliliter of blood will be collected from each participant in each tube separately following the sequence: gray-antigen-free tube (null control), red tube-TB antigen (ESAT-6, CPF-10, TB7.7) and purple tube-with hemagglutinin (mitogen or positive control). The tubes will be shared 10 times to contact the blood with the antigens.
2. Sample processing: Samples were incubated within a period of up to 1 hour after collection of blood. The tubes will be gently homogenized by inversion and taken to the oven at 37 ° C and placed vertically for 16 to 24 hours for incubation.
3. After incubation, the tubes will be centrifuged for 15 minutes between 2000 and 3000 RPM for plasma separation. The removed plasma will be placed in cryotubes and then frozen at minus 70 ° for further performance of the second phase of the technique. The cryotubes will be stored with the same identification made on the collection in separately identified trays (SAMPLE 1, SAMPLE 2).
4. The Elisa will be held only after the end of the inclusion period. The exams will be carried out in duplicate.

The regents except the 100X conjugate concentrate will be placed at room temperature for 60 minutes (min). During this time, the standard concentration was 8.0 IU / ml, and the conjugate 100X concentrated, both in distilled water. After reconstitution, the standard will be diluted using the diluent available in the kit: 4 IU / ml, 1UI / ml, 0.25 IU / ml, 0 IU / ml (in the latter, only green diluent).

After the dilutions were performed, 50μL of conjugate will be distributed in each well of the microtiter plate, then 50μL of plasma from the first test step was added to each well. This mixture was stirred for 1 min on a microplate shaker. At the end of this process, the plate containing the samples was covered to avoid exposure to light and incubated for 120 min at room temperature.

After dilution, 50μL of conjugate will be distributed in each well of the microtiter plate, then added 50μL of plasma to the first test step in each well. This mixture will be stirred for 1 min on a microplate shaker. At the end of this process, the plate containing the samples was covered to avoid exposure to light and incubated for 120 min at room temperature.

In the next step, the wells will be washed with 400 μl of the diluted wash buffer, available in the kit, which was repeated 6 times. Then 100 μl of the substrate will be added to each well and stirred for 1 min on the plate shaker. Again the plate will be covered and incubated for 30 min at room temperature. After that time, 50 μl of stop solution will be added to all wells and homogenized with the stirrer. Then the results will be read at 450nm with the reference filter of 620nm. The results that will be generated in the ELISA were analyzed in the software QuantiFERON-TB Gold Analysis and interpreted.

C) Interpretation of results:

| **Interpretação** | **Nil*** | **Antigen** | **Mitogen** |
| --- | --- | --- | --- |
| Interpretation | ≤8.0 | ≥0.35 IU/ml and ≥25% of Nil | Any |
| Positive | ≤8.0 | <0.35 IU/ml or <25% of Nil | ≥0.5 |
| Negative | ≤8.0 | <0.35 IU/ml or <25% of Nil | <0.5 |
|  | >8.0 | Any | Any |

Source: CELLESTIS, 2015

**4.3.4 Tuberculin Skin Test**

The tuberculin test will be performed by the intradermal application of 2 UT of purified protein derivative (PPD Rt 23) preferably on the inner surface of the left forearm at the first visit and preferably at the right at the second visit. If there is a psoriasis lesion at the site of application, the arms may be inverted. The results will be read after 72 hours and recorded in millimeters.

#### Data analysis plan

The IBM Statistical Package version 23.0 (IBM corp., Armonk, NY) will be used for the statistical calculations.

- Data will be analyzed descriptively through statistical measures: mean ± standard deviation or median and percentiles for numerical variables and absolute and percentage frequencies for categorical variables.
- It will be verified whether there is a significant change in the results of TST and/or IGRA tests, with 2 or 3 categories, classified according to the variables descriptions by the McNemar test. A P < 0,05 will be considered significant.
- It will be verified whether the difference in the comparison of test results is significant before and/ or after treatment by the McNemar test. A P < 0,05 will be considered significant.
- The agreement between each and between the IGRA and the TST before and after treatment, with two or three categories, will be verified by the Cohen’s kappa.
- For numeric continuous variables, the normality will be tested using the Shapiro-Wilk test. To compare the mean of paired continuous variables will be used the Student's t-test for paired variables for variables with a normal distribution, and the Wilcoxon test for variables with a non-normal distribution.
- To compare the TST and IGRA results with the means of numerical continuous variables will be used the Student's t-test for variables with a normal distribution, and the Mann-Whitney test for variables with a non-normal distribution.
- Will be verified the association between the sustainers or non-sustainers status with the categorized variables by the Fisher Exact test. The variables presenting p < 0.05 will be considered a positive association. The variables will be sex, age categorized, duration of illness categorized, alcohol consumption, smoking, presence of other forms of psoriasis associated, blood glucose (normal, diabetic).
- The association between the sustainers or non-sustainers status with the medians/medians of the continuous variables will be verified by t-Student test or Mann-Whitney test, The variables will be the age in years, disease duration in years, BMI, blood glucose in mg/dl, serum levels of INF-γ, TNF-α, interleukin-12, interleukin-17, interleukin-23, ESR, CPR. The association with ESR, CPR, and cytokines will be verified before and after treatment.
- The variables presenting p < 0.05 will be considered a positive association.

.

1. ***Budget****

| **ESPECIFICAÇÃO** | **EMBALAGEM** | **QNT** | **R$ UN** | **R$ TOTAL** | **ORIGEM** |
| --- | --- | --- | --- | --- | --- |
| **QFT Tubes** | 100 tubes | 1 | 3.099,25 | 3.099,25 | QIAGEN Brasil |
| **QFT 2 Plate Kit ELISA** | 96 tests | 2 | 1984,90 | 3968,00 | QIAGEN Brasil |
| **KIT ELISA IL-12** | 40 tests | 2 | 1.642,00 | 3.284,00 | INVITROGEN |
| **KIT-ELISA IL-23** | 40 tests | 2 | 1.642,00 | 3.284,00 | INVITROGEN |
| **KIT ELISA IL-17** | 40 tests | 2 | 1.642,00 | 3.284,00 | INVITROGEN |
| **KIT ELISA TNF-α** | 40 tests | 2 | 1.642,00 | 3.284,00 | INVITROGEN |
| **KIT ELISA INF-γ** | 40 tests | 2 | 1.642,00 | 3.284,00 | INVITROGEN |
| **Teste Tuberculínico** | 15 tests | 7 | 199,00 | 1.393,00 | Vaccines Rio |
| **Vacuum needle** | 100 Un | 2 | 38,50 | 77,00 | BD |
| **Hemo tube 0.3 ml vacuum collection** | 100 Un | 2 | 68,00 | 136,00 | BD |
| **Vacutainer Vacuum Blood Collection Adapter** | Un | 100 | 0,35 | 70,00 | BD |
| **Total** | | | | **R$ 25,163.25** | |

* The Project will be submitted to the public agencies of the research promotion agencies, as well as sponsorship will be sought from the pharmaceutical industry. The researcher will be responsible for the costs if there is no sponsorship to carry out the research.

1. ***References***

ABREU, C.; MAGRO, F.; SANTOS-ANTUNES, J.; PILÃO, A.; RODRIGUES-PINTO, E.; BERNARDES, J.; BERNARDO, A.; MAGINA, S.; VILAS-BOAS, F.; LOPES, S.; MACEDO, G.; SARMENTO, A. J. Tuberculosis in anti-TNF-α treated patients remains a problem in countries with an intermediate incidence: Analysis of 25 patients matched with a control population. Journal of Crohn's and colitis, Amsterdam, v. 7, n.10, p. 486-92, 2013.

BASSUKAS, I. D.; KOSMIDOU, M.; GAITANIS, G.; TSIOURI, G.; TSIANOS, E. Patients with psoriasis are more likely to be treated for latent tuberculosis infection before biologics than patients with inflammatory bowel disease. Acta Dermato-Venereologica, Estocolmo, v. 91, n. 4, p. 444-6, 2011.

BARTALESI, F.; VICIDOMINI, S.; GOLETTI, D. et al. QuantiFERON-TB Gold and the TST are both useful for latent tuberculosis infection screening in autoimmune diseases. The European respiratory journal, Copenhagen, v. 33, n.3, p. 586–593, 2009.

CONSENSO BRASILEIRO DE PSORÍASE 2012 - Guias de avaliação e tratamento Sociedade Brasileira de Dermatologia. – 2 ed. Rio de Janeiro: Sociedade Brasileira de Dermatologia, 2009. 172 p.; 1 ed.; 24 pg.

COMISSÃO DE TUBERCULOSE DA SBPT, GRUPO DE TRABALHO DAS DIRETRIZES PARA TUBERCULOSE DA SBPT. III Diretrizes para Tuberculose da Sociedade Brasileira de Pneumologia e Tisiologia. Jornal Brasileiro de Pneumologia, Brasilia, v. 35, n. 10, p. 1018-1048, 2009.

[COSTANTINO, F](http://www.ncbi.nlm.nih.gov/pubmed?term=Costantino%20F%5BAuthor%5D&cauthor=true&cauthor_uid=24085550).; [DE CARVALHO BITTENCOURT, M](http://www.ncbi.nlm.nih.gov/pubmed?term=de%20Carvalho%20Bittencourt%20M%5BAuthor%5D&cauthor=true&cauthor_uid=24085550).; [RAT, A. C](http://www.ncbi.nlm.nih.gov/pubmed?term=Rat%20AC%5BAuthor%5D&cauthor=true&cauthor_uid=24085550).; [LOEUILLE, D](http://www.ncbi.nlm.nih.gov/pubmed?term=Loeuille%20D%5BAuthor%5D&cauthor=true&cauthor_uid=24085550).; [DINTINGER, H](http://www.ncbi.nlm.nih.gov/pubmed?term=Dintinger%20H%5BAuthor%5D&cauthor=true&cauthor_uid=24085550).; [BÉNÉ, M. C](http://www.ncbi.nlm.nih.gov/pubmed?term=B%C3%A9n%C3%A9%20MC%5BAuthor%5D&cauthor=true&cauthor_uid=24085550).; [FAURE, G](http://www.ncbi.nlm.nih.gov/pubmed?term=Faure%20G%5BAuthor%5D&cauthor=true&cauthor_uid=24085550).; CHARY-VALCKENAERE, I. Screening for Latent Tuberculosis Infection in Patients with Chronic Inflammatory Arthritis: Discrepancies Between Tuberculin Skin Test and Interferon-γ Release Assay Results.  [The Journal of rheumatology, Toronto](http://www.ncbi.nlm.nih.gov/pubmed/24085550), v. 40, n. 12, p. 1986-93, 2013.

[DAUDÉN, E](https://www.ncbi.nlm.nih.gov/pubmed/?term=Daud%C3%A9n%20E%5BAuthor%5D&cauthor=true&cauthor_uid=26812550).; [PUIG, L](https://www.ncbi.nlm.nih.gov/pubmed/?term=Puig%20L%5BAuthor%5D&cauthor=true&cauthor_uid=26812550).; [FERRÁNDIZ, C](https://www.ncbi.nlm.nih.gov/pubmed/?term=Ferr%C3%A1ndiz%20C%5BAuthor%5D&cauthor=true&cauthor_uid=26812550).; [SÁNCHEZ-CARAZO, J.L](https://www.ncbi.nlm.nih.gov/pubmed/?term=S%C3%A1nchez-Carazo%20JL%5BAuthor%5D&cauthor=true&cauthor_uid=26812550).; [HERNANZ-HERMOSA, J.M](https://www.ncbi.nlm.nih.gov/pubmed/?term=Hernanz-Hermosa%20JM%5BAuthor%5D&cauthor=true&cauthor_uid=26812550).; [SPANISH PSORIASIS GROUP OF THE SPANISH ACADEMY OF DERMATOLOGY AND VENEREOLOGY](https://www.ncbi.nlm.nih.gov/pubmed/?term=Spanish%20Psoriasis%20Group%20of%20the%20Spanish%20Academy%20of%20Dermatology%20and%20Venereology%5BCorporate%20Author%5D). Consensus document on the evaluation and treatment of moderate-to-severe psoriasis: Psoriasis Group of the Spanish Academy of Dermatology and Venereology. Journal of European Academy of Dermatology and Venereology, Amsterdam, v.30, n. Suppl 2, pg. 1-18. 2016.

DE ANDRADE LIMA, E.; DE ANDRADE LIMA, M.; DE LORENA, V. M.; DE MIRANDA, GOMES, Y.; LUPI, O.; BENARD, G. Evaluation of an IFN-gamma assay in the diagnosis of latent tuberculosis in patients with psoriasis in a highly endemic setting. Acta Dermato-Venereologica, Estocolmo, v. 91, n.6, p. 694-7, 2011.

DOHERTY, S. D.; VAN VOORHEES, A.; LEBWOHL, M. G.; KORMAN, N. J.; YOUNG, M. S.; HSU, S. National Psoriasis Foundation consensus statement on screening for latent tuberculosis infection in patients with psoriasis treated with systemic and biologic agents. Journal of the American Academy of Dermatology, St. Louis, v. 59, n. 2, p. 209-17, 2008.

FORTALEZA, G. T.; BRITO, DE F.; SANTOS, J. B.; FIGUEIREDO, A. R.; GOMES, P. [Splenic tuberculosis during psoriasis treatment with infliximab.](http://www.ncbi.nlm.nih.gov/pubmed/19851677) Anais Brasileiros de Dermatologia, Rio de Janeiro, v. 84, n. 4, p. 420-4, 2009.

GOUJON, C.; GORMAND, F.; GUNERA-SAAD, N.; DAHEL, K.; VIAL, T.; NICOLAS, J. F. The relevance of diagnostic criteria for latent tuberculosis before initiation of TNF-alpha inhibitors in psoriasis patients. Annales de dermatologie et de vénéréologie, Paris-New York, v. 137, n. 6-7, p. 437-43, 2010.

GRUPO DE TRABAJO DEL ÁREA TIR DE SEPAR. Recomendaciones
SEPAR; normativa sobre la prevención de la tuberculosis. Archivos de Bronconeumología, Barcelona v.38, n.9 p. 441-451, 2002

[HELWIG, U](http://www.ncbi.nlm.nih.gov/pubmed?term=Helwig%20U%5BAuthor%5D&cauthor=true&cauthor_uid=22398067).; [MÜLLER, M](http://www.ncbi.nlm.nih.gov/pubmed?term=M%C3%BCller%20M%5BAuthor%5D&cauthor=true&cauthor_uid=22398067).; [HEDDERICH, J](http://www.ncbi.nlm.nih.gov/pubmed?term=Hedderich%20J%5BAuthor%5D&cauthor=true&cauthor_uid=22398067).; [SCHREIBER, S](http://www.ncbi.nlm.nih.gov/pubmed?term=Schreiber%20S%5BAuthor%5D&cauthor=true&cauthor_uid=22398067). Corticosteroids, and immunosuppressive therapy influence the result of QuantiFERON TB Gold testing in inflammatory bowel disease patients.  [Journal of Crohn's and colitis, Amsterdam.](http://www.ncbi.nlm.nih.gov/pubmed/22398067)  v.6, n. 4, p. 419-24, 2012.

KARATAŞ TOĞRAL, A.; MUŞTU KORYÜREK, Ö.; ŞAHIN, M.; et al. Association of clinical properties and compatibility of the QuantiFERON-TB gold In-Tube test with the tuberculin skin test in patients with psoriasis. International journal of dermatology, Philadelphia v.55, n.6, pg. 629-33, 2016.

LAFFITTE, E.; JANSSENS, J. P.; ROUX-LOMBARD, P.; THIELEN, A. M.; BARDE, C.; MARAZZA, G.; PANIZZON, R.G.; SAURAT, J.H. Tuberculosis screening in patients with psoriasis before antitumor necrosis factor therapy: comparison of an interferon-gamma release assay vs. tuberculin skin test. British Journal of Dermatology, London, v. 161, n. 4, p. 797-800, 2009.

LATORRE I, CARRASCOSA JM, VILAVELLA M, DIAZ J, PRAT C, DOMINGUEZ J, et al. Diagnosis of tuberculosis infection by interferon-gamma release assays in patients with psoriasis**. Journal of Infection**. v. 69, n. 6, p. 600–606, 2014

MARTYN-SIMMONS, C.L.; MEE, J.B.; KIRKHAM, B.W.; GROVES, R.W.; MILBURN, H.J. Evaluating the use of the interferon-γ response to Mycobacterium tuberculosis-specific antigens in patients with psoriasis prior to antitumor necrosis factor-α therapy: a prospective head-to-head cross-sectional study. British Journal of Dermatology, London, v. 168, n. 5, p. 1012-8, 2013.

RAMOS, J.M.; MASIÁ, M.; RODRÍGUEZ, J.C.; LÓPEZ C.; PADILLA S.; ROBLEDANO, C.; NAVARRO-BLASCO, F.J.; MATARREDONA, J.; GARCÍA-SEPULCRE, M.F.; GUTIÉRREZ, F. Negative effect of immunosuppressive therapy in the performance of the QuantiFERON gold in-tube test in patients with immune-mediated inflammatory diseases. Clinical and Experimental Medicine, Milano, v. 177, n. 86, p. 177-86, 2013.

SAUZULLO, I.; MENGONI, F.; MAROCCO, R.; POTENZA, C.; SKROZA, N.; TIEGHI, T.; LICHTNER, M.; VULLO, V.; MASTROIANNI, C.M. [Interferon-γ release assay for tuberculosis in patients with psoriasis treated with tumor necrosis factor antagonists: in vivo and in vitro analysis.](http://www.ncbi.nlm.nih.gov/pubmed/23909256) British Journal of Dermatology, London, v.169, n.5, pg.1133-1140, 2013.

[SHAIKHA, S. A](http://www.ncbi.nlm.nih.gov/pubmed/?term=Shaikha%20SA%5BAuthor%5D&cauthor=true&cauthor_uid=22529801).; [MANSOUR, K](http://www.ncbi.nlm.nih.gov/pubmed/?term=Mansour%20K%5BAuthor%5D&cauthor=true&cauthor_uid=22529801).; HASSAN, [R. Reactivation of Tuberculosis in Three Cases of Psoriasis after Initiation of Anti-TNF Therapy. [Case Reports in Dermatology, Basel,](http://www.ncbi.nlm.nih.gov/pubmed/?term=Reactivation+of+Tuberculosis+in+Three+Cases+of+Psoriasis+after+Initiation+of+Anti-TNF+Therapy)  v. 4, n.1, p. 41-6,](http://www.ncbi.nlm.nih.gov/pubmed/?term=Riad%20H%5BAuthor%5D&cauthor=true&cauthor_uid=22529801)  2012.

SOLOVIC, I.; SESTER, M.; GOMEZ-REINO, J.J.; RIEDER, H.L.; EHLERS, S.; MILBURN, H.J.; KAMPMANN, B.; HELLMICH, B.; GROVES, R.; SCHREIBER, S.; WALLIS, R.S.; SOTGIU, G.; SCHÖLVINCK, E.H.; GOLETTI, D.; ZELLWEGER, J,P.; DIEL, R.; CARMONA, L.; BARTALESI, F.; RAVN, P.; BOSSINK, A.; DUARTE, R.; ERKENS, C.; CLARK, J.; MIGLIORI, G.B.; LANGE, C. The risk of tuberculosis related to tumor necrosis factor antagonist therapies: a TBNET consensus statement. The European respiratory journal, Copenhague, v. 36, n. 5, p.1185-206, 2010.

TAVAST, E.; TUUMINEN, T.; PAKKANEN, S.H.; ERIKSSON, M.; KANTELE, A.; JÄRVINEN, A.; PUSA, L.; MÄLKÖNEN, T.; SEPPÄLÄ, I.; REPO, H.; LERISALO-REPO, M. [Immunosuppression Adversely Affects TST but Not IGRAs in Patients with Psoriasis or Inflammatory Musculoskeletal Diseases.](http://www.ncbi.nlm.nih.gov/pubmed/22666260) International Journal of rheumatology, New York, 381929, 2012.

TSIOURI, G.; GAITANIS, G.; KIORPELIDOU, D.; DIONYSIOU, A.; EFTHYMIOU, A.; DASKALOPOULOS. G.; CONSTANTOPOULOS, S.; BASSUKAS, I.D. Tuberculin skin test overestimates tuberculosis hypersensitivity in adult patients with psoriasis. Dermatology. Basel-New York, v. 219, n. 2, p. 119-25, 2009.

[YOO, I.K](http://www.ncbi.nlm.nih.gov/pubmed?term=Yoo%20IK%5BAuthor%5D&cauthor=true&cauthor_uid=24532516).; [CHOUNG, R.S](http://www.ncbi.nlm.nih.gov/pubmed?term=Choung%20RS%5BAuthor%5D&cauthor=true&cauthor_uid=24532516).; [HYUN, J.J](http://www.ncbi.nlm.nih.gov/pubmed?term=Hyun%20JJ%5BAuthor%5D&cauthor=true&cauthor_uid=24532516).; [KIM, S.Y](http://www.ncbi.nlm.nih.gov/pubmed?term=Kim%20SY%5BAuthor%5D&cauthor=true&cauthor_uid=24532516).; [JUNG, S.W](http://www.ncbi.nlm.nih.gov/pubmed?term=Jung%20SW%5BAuthor%5D&cauthor=true&cauthor_uid=24532516).; [KOO, J.S](http://www.ncbi.nlm.nih.gov/pubmed?term=Koo%20JS%5BAuthor%5D&cauthor=true&cauthor_uid=24532516).; [LEE, S.W](http://www.ncbi.nlm.nih.gov/pubmed?term=Lee%20SW%5BAuthor%5D&cauthor=true&cauthor_uid=24532516).; [CHOI, J.H](http://www.ncbi.nlm.nih.gov/pubmed?term=Choi%20JH%5BAuthor%5D&cauthor=true&cauthor_uid=24532516).; [KIM, H](http://www.ncbi.nlm.nih.gov/pubmed?term=Kim%20H%5BAuthor%5D&cauthor=true&cauthor_uid=24532516).; [LEE, H.S](http://www.ncbi.nlm.nih.gov/pubmed?term=Lee%20HS%5BAuthor%5D&cauthor=true&cauthor_uid=24532516).; [KEUM, B](http://www.ncbi.nlm.nih.gov/pubmed?term=Keum%20B%5BAuthor%5D&cauthor=true&cauthor_uid=24532516).; [KIM, E.S](http://www.ncbi.nlm.nih.gov/pubmed?term=Kim%20ES%5BAuthor%5D&cauthor=true&cauthor_uid=24532516).; [JEEN, Y.T](http://www.ncbi.nlm.nih.gov/pubmed?term=Jeen%20YT%5BAuthor%5D&cauthor=true&cauthor_uid=24532516). Incidences of serious infections and tuberculosis among patients receiving anti-tumor necrosis factor-α therapy. [Yonsei Medical Journal. Seul.](http://www.ncbi.nlm.nih.gov/pubmed/24532516)  v.55, n. 2, p.442-8, 2014.

1. ***Appendices***

**Appendix 4 - Patient free and informed consent form**

**UFPE - TERMO DE CONSENTIMENTO LIVRE E ESCLARECIDO (PARA MAIORES DE 18 ANOS OU EMANCIPADOS - Resolução 466/12)**

Convidamos o (a) Sr. (a) para participar como voluntário (a) da pesquisa “**Interferência do metotrexato sobre o QuantiFERON Tb-Gold na triagem de tuberculose latente e sua relação com a atividade clínico-imunológica da psoríase,** que está sob a responsabilidade do (a) pesquisador (a) **VANESSA LUCÍLIA SILVEIRA DE MEDEIROS**, residente á Av. Engenheiro Domingos Ferreira 3856, AP 302, Boa viagem, Recife, PE, CEP 51021-040, telefone de contato 32042840, e-mail [dermatorecife@yahoo.com.br](mailto:dermatorecife@yahoo.com.br) e está sob a orientação de **VERA MAGALHÃES DA SILVEIRA**, Telefones para contato: 81 21268527, e-mail vemagalhães@uol.com.br.

Este Termo de Consentimento pode conter alguns tópicos que o/a senhor/a não entenda. Caso haja alguma dúvida, pergunte à pessoa a quem está lhe entrevistando, para que o/a senhor/a esteja bem esclarecido (a) sobre tudo que está respondendo. Após ser esclarecido (a) sobre as informações a seguir, caso aceite em fazer parte do estudo, rubrique as folhas e assine ao final deste documento, que está em duas vias. Uma delas é sua e a outra é do pesquisador responsável. Em caso de recusa o (a) Sr. (a) não será penalizado (a) de forma alguma. Também garantimos que o (a) Senhor (a) tem o direito de retirar o consentimento da sua participação em qualquer fase da pesquisa, sem qualquer penalidade.

**INFORMAÇÕES SOBRE A PESQUISA:**

⮚Essa pesquisa busca saber se um dos medicamentos utilizados para tratar a psoríase (metotrexato) atrapalha o resultado de um exame para diagnóstico de tuberculose. Também quer saber se esse exame é alterado pela gravidade da doença. Para saber isso é necessário fazer o exame físico completo, retirar sangue para o teste da tuberculose, para exames imunológicos, para exames gerais e fazer Rx de tórax para verificar se tem tuberculose ou não. Após 3 meses de uso do metotrexato a consulta e os exames de sangue serão repetidos. Essa rotina é praticada para todos os pacientes mesmo fora da pesquisa que estão tomando metotrexato. O único teste a mais a ser realizado é da tuberculose

⮚O período de participação na pesquisa é de 3 meses com período posterior de seguimento por tempo indeterminado. Esse é o período normal entre duas consultas de pacientes que estão usando metotrexato. O seu tratamento ocorrerá normalmente após esse período. No ambulatório de psoríase.

Durante a consulta o paciente deverá retirar a roupa para ser examinado, e assim o médico possa verificar o quanto a psoríase é grave. Você pode se sentir mal por isso, porém o exame é feito dessa forma mesmo em quem não está participando da pesquisa. Você irá sentir um pouco de dor durante a retirada de sangue. Normalmente a dor passa imediatamente. Pode ficar roxo no loca da picada que passa em 7 dias.

⮚ Com os resultados dos exames você saberá se teve contato a tuberculose, se tem hepatite ou AIDS e ajudará a esclarecer se esse teste para tuberculose é bom para pacientes com psoríase.

As informações desta pesquisa serão confidencias e serão divulgadas apenas em eventos ou publicações científicas, não havendo identificação dos voluntários, a não ser entre os responsáveis pelo estudo, sendo assegurado o sigilo sobre a sua participação. Os dados coletados nesta pesquisa (ficha de coleta de dados) ficarão armazenados em pastas de arquivo, sob a responsabilidade do pesquisador, no endereço acima informado pelo período de (mínimo 5 anos).

O (a) senhor (a) não pagará nada para participar desta pesquisa. Se houver necessidade, as despesas para a sua participação serão assumidos pelos pesquisadores (ressarcimento de transporte e alimentação). Fica também garantida indenização em casos de danos, comprovadamente decorrentes da participação na pesquisa, conforme decisão judicial ou extra-judicial.

Em caso de dúvidas relacionadas aos aspectos éticos deste estudo, você poderá consultar o Comitê de Ética em Pesquisa Envolvendo Seres Humanos da UFPE no endereço: **(Avenida da Engenharia s/n – 1º Andar, sala 4 - Cidade Universitária, Recife-PE, CEP: 50740-600, Tel.: (81) 2126.8588 – e-mail:** [**cepccs@ufpe.br**](mailto:cepccs@ufpe.br)**).**

___________________________________________________

(assinatura do pesquisador)

**CONSENTIMENTO DA PARTICIPAÇÃO DA PESSOA COMO VOLUNTÁRIO (A)**

Eu, _____________________________________, CPF _________________, abaixo assinado, após a leitura (ou a escuta da leitura) deste documento e de ter tido a oportunidade de conversar e ter esclarecido as minhas dúvidas com o pesquisador responsável, concordo em participar do estudo “**Interferência do metotrexato sobre o QuantiFERON Tb-Gold na triagem de tuberculose latente e sua relação com a atividade clínico-imunológica da psoríase,”**, como voluntário (a). Fui devidamente informado (a) e esclarecido (a) pelo(a) pesquisador (a) sobre a pesquisa, os procedimentos nela envolvidos, assim como os possíveis riscos e benefícios decorrentes de minha participação. Foi-me garantido que posso retirar o meu consentimento a qualquer momento, sem que isto leve a qualquer penalidade (ou interrupção de meu acompanhamento/ assistência/tratamento).

Impressão digital (opcional)

Local e data __________________

Assinatura do participante: __________________________

**Presenciamos a solicitação de consentimento, esclarecimentos sobre a pesquisa e o aceite do voluntário em participar. (**02 testemunhas não ligadas à equipe de pesquisadores):

| Nome: | Nome: |
| --- | --- |
| Assinatura: | Assinatura: |

**Appendix 2 - Clinical Data Record**

| CLINICAL DATA RECORD | | | | | |
| --- | --- | --- | --- | --- | --- |
| Name | | | | Initials: | |
| Birthdate | | | | Research Number | |
| Date of onset of illness: | | | | Sex   1. Male 2. Female | |
| Current Medicine intake: | | | | | |
| BCG scar  Current or less than 6 months use of topical treatment  Previous use of immunosuppressant  Current treatment for psoriasis  Symptoms of active TB  Complete or prophylactic treatment for TB  Laboratory tests with contraindication to methotrexate  In case of yes, describe:  Breastfeeding: | | | 1. Yes 2. No  1. Yes 2. No  1. Yes 2. No  1. Yes 2. No  1. Yes 2. No  1. Yes 2. No  1. Yes 2. No  1. Yes 2. No | | |
| Chest x-ray result description:  Normal 1. Altered 2.  alteration non-LTBI 1. Yes 2. No | | | | | |
| Glycemia in mg/dl |  | Scalp psoriasis | | | 1. Yes 2. No |
| Contact with TB carrier | 1. Yes 2. No | Geographic tongue | | | 1. Yes 2. No |
| Residence place | 1. Urban 2. Rural | Nail psoriasis | | | 1. Yes 2. No |
| Contact with cattle | 1. Yes 2. No | Inverted psoriasis | | | 1. Yes 2. No |
| Alcohol intake | 1. Yes 2. No | Palmoplantar psoriasis | | | 1. Yes 2. No |
| Smoking | 1. Yes 2. No | Enthesitis | | | 1. Yes 2. No |
| Diabetes | 1. Yes 2. No | Scalp psoriasis | | | 1. Yes 2. No |

**Appendix 3- Standardized questionnaire for screening for tuberculosis**

| **Initials** | | **Research number** |
| --- | --- | --- |
| **The patient has presented some symptom compatible with TB:** | | |
| 1 | Productive cough for more than 3 weeks | 1. Yes 2. No |
| 2 | Hemoptysis | 1. Yes 2. No |
| 3 | Asthenia | 1. Yes 2. No |
| 4 | Fever | 1. Yes 2. No |
| 5 | Night sweats | 1. Yes 2. No |
| 6 | Anorexia | 1. Yes 2. No |
| 7 | Weight loss over 10% of ideal weight | 1. Yes 2. No |
| If any of the above answers were positive, active TB needs to be completely discarded. | | |
| **The patient has presented other risk factors for TBLI** | | |
| 8 | Diabetes | 1. Yes 2. No |
| 9 | Illicit drug use | 1. Yes 2. No |
| 10 | Born in an endemic area for TB | 1. Yes |
| 11 | History of previous treatment for TB | 1. Yes 2. No |
| 12 | History of previous prophylaxis for TB | 1. Yes 2. No |
| 13 | Family history of TB |  |
| A | Parents | 1. Yes 2. No |
| B | Grandparents | 1. Yes 2. No |
| C | Partner | 1. Yes 2. No |
| D | Child | 1. Yes 2. No |
| E | Own patient | 1. Yes 2. No |
| 14 | Recent contact with TB carrier | 1. Yes 2. No |
| 15 | Live or work in a high-risk environment | 1. Yes 2. No |

**Appendix 4 - Laboratory Follow-up Record**

| **Flowchart of laboratory exams** | | | | | | | | | | | |
| --- | --- | --- | --- | --- | --- | --- | --- | --- | --- | --- | --- |
| **Initials** | | | | | | | **Research number** | | | |  |
| **DATE** |  |  |  |  |  |  |  |  |  |  |  |
| **HB** |  |  |  |  |  |  |  |  |  |  |  |
| **HT** |  |  |  |  |  |  |  |  |  |  |  |
| **VCM** |  |  |  |  |  |  |  |  |  |  |  |
| **HCM** |  |  |  |  |  |  |  |  |  |  |  |
| **LEUCO** |  |  |  |  |  |  |  |  |  |  |  |
| **SEG** |  |  |  |  |  |  |  |  |  |  |  |
| **INF** |  |  |  |  |  |  |  |  |  |  |  |
| **EOS** |  |  |  |  |  |  |  |  |  |  |  |
| **MONO** |  |  |  |  |  |  |  |  |  |  |  |
| **PLT** |  |  |  |  |  |  |  |  |  |  |  |
| **UREIA** |  |  |  |  |  |  |  |  |  |  |  |
| **CREA** |  |  |  |  |  |  |  |  |  |  |  |
| **AST** |  |  |  |  |  |  |  |  |  |  |  |
| **ALT** |  |  |  |  |  |  |  |  |  |  |  |
| **FALC** |  |  |  |  |  |  |  |  |  |  |  |
| **GGT** |  |  |  |  |  |  |  |  |  |  |  |
| **Total cholesterol** |  |  |  |  |  |  |  |  |  |  |  |
| **HDL** |  |  |  |  |  |  |  |  |  |  |  |
| **LDL** |  |  |  |  |  |  |  |  |  |  |  |
| **VLDL** |  |  |  |  |  |  |  |  |  |  |  |
| **TRIGLI** |  |  |  |  |  |  |  |  |  |  |  |
| **GLI** |  |  |  |  |  |  |  |  |  |  |  |
| **ESR** |  |  |  |  |  |  |  |  |  |  |  |
| **CPR** |  |  |  |  |  |  |  |  |  |  |  |
| **Serologies** |  |  |  |  |  |  |  |  |  |  |  |
| **Others** |  |  |  |  |  |  |  |  |  |  |  |
| **Medication** |  |  |  |  |  |  |  |  |  |  |  |

**Appendix 5 – PASI SCORE CALCULATION**

| PASI | | | | |
| --- | --- | --- | --- | --- |
| Initials | | | Research number | |
|  | Head | | Arms | |
| Area in% |  0  <10   10-20  30-49    50-69  70-89 90-100 | |  0 <10   10-20 30-49    50-69  70-89 90-100 | |
| Erythema | 0 1 2 3 4 | | 0 1 2 3 4 | |
| Thickness | 0 1 2 3 4 | | 0 1 2 3 4 | |
| Desquamation | 0 1 2 3 4 | | 0 1 2 3 4 | |
|  | Trunk | | Legs | |
| Area in% |  0  <10   10-20  30-49    50-69  70-89 90-100 | | 0 <10   10-20 30-49   50-69 70-8990-100 | |
| Erythema | 0 1 2 3 4 | | 0 1 2 3 4 | |
| Thickness | 0 1 2 3 4 | | 0 1 2 3 4 | |
| Desquamation | 0 1 2 3 4 | | 0 1 2 3 4 | |
|  | Head x 0,1 |  | Arms x 0,3 |  |
|  | Trunk x 0,3 |  | Legs x 0,4 |  |
| Total |  | |  | |

**Appendix 6- Laboratory Data Record**

| **LABORATORY DATA RECORD** | |
| --- | --- |
| **Initials** | **Research number** |
| **Birthdate** | **Sex** |

| Test | Result Before MTX | Result After MTX |
| --- | --- | --- |
| QuantiFERON TB-Gold |  |  |
| TNF-alfa in pg/ml |  |  |
| INF-Gama in pg/ml |  |  |
| IL17 in pg/ml |  |  |
| IL 12 in pg/ml |  |  |
| IL23 in pg/ml |  |  |
| CRP in mg/dl |  |  |
| ESR in mm/h |  |  |
| Tuberculin skin test |  |  |

**Appendix 7- Hospital Consent to carry out the study**

**Appendix 8-Term of data confidentiality**

**Appendix 9- Authorization to access patient data**
