# Supplementary material for: TST conversions and systemic interferon-gamma increase after methotrexate introduction in psoriasis patients
Source: PLoS One. 2020 Dec 3;15(12):e0242098. doi: 10.1371/journal.pone.0242098 (PMC7714364; doi:10.1371/journal.pone.0242098)
Supplement: Attachment — Submitted filename: Reviews answer.docx [file pone.0242098.s008.docx]

Response to Reviewers letter

**Comments to the Author**

1. Is the manuscript technically sound, and do the data support the conclusions?

The manuscript must describe a technically sound piece of scientific research with data that supports the conclusions. Experiments must have been conducted rigorously, with appropriate controls, replication, and sample sizes. The conclusions must be drawn appropriately based on the data presented.

Reviewer #1: Partly

Reviewer #2: Partly

***Answer:*** *The manuscript was rewritten modifying the view of the data found. Since its submission, new studies reinforce the findings.*

2. Has the statistical analysis been performed appropriately and rigorously?

Reviewer #1: No

Reviewer #2: I Don't Know

***Answer****: The sample size was calculated based on the formula for paired variables and was added to the text. It seems that the N is small, but it is even above the minimum required. The calculation can be easily performed in standard programs. Similar studies have small N as well. The largest study is retrospective, with 70 patients, but 90% with negative results for both tests ^1^. My study have proportionally more positive results and being a study type before and after, requires a smaller sample.*

1. Martyn-Simmons CL, Mee JB, Kirkham BW, Groves RW, Milburn HJ. Evaluating the use of the interferon-gamma response to *Mycobacterium tuberculosis*-specific antigens in patients with psoriasis prior to antitumour necrosis factor-alpha therapy: a prospective head-to-head cross-sectional study. Br J Dermatol. 2013;168: 1012-1018.

***Answer*** *: Calculations of the IGRA and TST change were performed for both 2 categories (binary) and 3 ( positive, indeterminate and negative) categories. The calculations can be performed from the results presented in the complete table 3 submitted as supplementary material. The tests were compared in the same way. The results showed no differences in the two forms of calculation. The calculation of associations with continuous variables showed no association, except for IGRA and ESR. Performed with 3 categories there will be no modification in these results and the association with the ESR will be lost because N of each group will be very small. Therefore, the fact that the test evaluation was performed in two or three categories did not impact in truly important results.*

3. Have the authors made all data underlying the findings in their manuscript fully available?

The [PLOS Data policy](http://www.plosone.org/static/policies.action#sharing) requires authors to make all data underlying the findings described in their manuscript fully available without restriction, with rare exception (please refer to the Data Availability Statement in the manuscript PDF file). The data should be provided as part of the manuscript or its supporting information, or deposited to a public repository. For example, in addition to summary statistics, the data points behind means, medians and variance measures should be available. If there are restrictions on publicly sharing data—e.g. participant privacy or use of data from a third party—those must be specified.

Reviewer #1: Yes

Reviewer #2: No

***Answer****: All data were shared in the supplementary material, except for completed table 3, which I am adding now. The results were summarized in a short table due to data redundancy and the extension of the tables. For the same reason, the text presented a summary of the data in tables 5,6,7, and 8. If necessary, I can share the complete database in excel if necessary. The same data can be acessed is in a public repository :sucupira.capes.gov.br/sucupira/public/consultas/coleta/trabalhoConclusao/viewTrabalhoConclusao.jsf?popup=true&id_trabalho=6291465*

4. Is the manuscript presented in an intelligible fashion and written in standard English?

PLOS ONE does not copyedit accepted manuscripts, so the language in submitted articles must be clear, correct, and unambiguous. Any typographical or grammatical errors should be corrected at revision, so please note any specific errors here.

Reviewer #1: Yes

Reviewer #2: No

 ***Answer****:* *The manuscript was sent for an English review and presents a certificate of the process. I apologize for the incorrect texts*

5. Review Comments to the Author

Please use the space provided to explain your answers to the questions above. You may also include additional comments for the author, including concerns about dual publication, research ethics, or publication ethics. (Please upload your review as an attachment if it exceeds 20,000 characters)

Reviewer #1: This manuscript tends to compare tuberculin Skin test (TST) and interferon-Gamma release assay (IGRA) in determining prognostic on Methotrexate-based psoriasis treatment. The title of this manuscript does not match the contents in the text. The Introduction is confusing about the reasons of this study. The 1st paragraph of introduction indicates that TST has both false negative and higher false positive results. The 3rd paragraph said that IGRA appears to superior to the TST but some studies have found increased numbers of negative and indeterminate IGRA results in immunosuppressed patients. It sounds that this study will compare which test (TST or IGRA) will have more accurate testing results about LTBI. But the last two paragraphs of Introduction changed to different questions about whether MTX treatment would affect the screening tests. It is not clear why do TST or IGRA test. Is it for screening LTBI or try to use them to predict prognostic on Methotrexate-based psoriasis treatment?

**Answer:**  *I changed the title and the discussion*. *The main propose of the study is to verify the changes of IGRA and TST after the use of MTX*

How was the sample size determined for this study? What is the main endpoint?- ***Answered on the questions 1 and 2***

Line 154, “tree” should be “three”.- ***Corrected***

Lines 197-198, what do bicaudal for IGRA and monocaudal for TST mean? What’s bicaudal McNemar test?

**Answer**  *The TST changes only for positive. Unicaldal means one tail, In other words, change only in one direction. IGRA presented changes for both sides, so the test has two tails. To avoid distortions in the result, a correction is applied according to the number of tails .This is called Yates' correction. It is not the author's choice. It is an automatic input of the calculation exactly to avoid distortions. I removed this from the text so as not to confuse the reader.*

Line 201, it is not clear when will use a t-test and paired t-test?

**Answer**  *T-Test or was used for the associations between IGRA or TST and the clinical and laboratory continuous variables. The paired T-test or Wilcoxon were used to analyze changes in the means of paired continuous variables ( inflammatory markers and the PASI). I corrected in the text to make it clear*

Line 202, it is not clear which Wilcoxon test do you indicate in “Wilcoxon or Mann-whitney test”?

***Answer:*** *I corrected in the text to make it clear.*

Line 238, there are not these numbers in Table 3

***Answer:*** *There was a mistake in the second case. I corrected the number*

Line 239-242, No clues about what these sentences mean. E.g. What data could be used to confirm and what are the possibility of analyzing indeterminate results along positive results?

***Answer*** *IGRA showed agreement within the same range with two or three variables, so there is no difference in analyzing the results in one way or another. Other authors also support that the indeterminate results are mostly due to positive results and so should be considered. ^2^*

2. [*Uzorka JW*](https://www.ncbi.nlm.nih.gov/pubmed/?term=Uzorka%20JW%5BAuthor%5D&cauthor=true&cauthor_uid=29146607)*,* [*Kroft LJM*](https://www.ncbi.nlm.nih.gov/pubmed/?term=Kroft%20LJM%5BAuthor%5D&cauthor=true&cauthor_uid=29146607)*,* [*Bakker JA*](https://www.ncbi.nlm.nih.gov/pubmed/?term=Bakker%20JA%5BAuthor%5D&cauthor=true&cauthor_uid=29146607)*,* [*van Zwet EW*](https://www.ncbi.nlm.nih.gov/pubmed/?term=van%20Zwet%20EW%5BAuthor%5D&cauthor=true&cauthor_uid=29146607)*,* [*Huisman E*](https://www.ncbi.nlm.nih.gov/pubmed/?term=Huisman%20E%5BAuthor%5D&cauthor=true&cauthor_uid=29146607)*, [Knetsch-Prins C](https://www.ncbi.nlm.nih.gov/pubmed/?term=Knetsch-Prins%20C%5BAuthor%5D&cauthor=true&cauthor_uid=29146607),* [*van der Zwan CJ*](https://www.ncbi.nlm.nih.gov/pubmed/?term=van%20der%20Zwan%20CJ%5BAuthor%5D&cauthor=true&cauthor_uid=29146607)*,  [Ottenhoff THM](https://www.ncbi.nlm.nih.gov/pubmed/?term=Ottenhoff%20THM%5BAuthor%5D&cauthor=true&cauthor_uid=29146607),* [*Arend SM*](https://www.ncbi.nlm.nih.gov/pubmed/?term=Arend%20SM%5BAuthor%5D&cauthor=true&cauthor_uid=29146607)*. Proof of concept that most borderline Quantiferon results are true antigen-Specific responses.* [*Eur Respir J.*](https://www.ncbi.nlm.nih.gov/pubmed/?term=Proof+of+concept+that+most+borderline+Quantiferon+results+are+true+antigen-specific+responses) *2017 Nov 16;50(5).*

Table 3, What’s the meaning to test the agreement for each test between before and after MTX treatment without consideration of potential change in LTBI condition? Any gold test could be used as reference?

***Answer:*** *There is no gold standard for LTBI screening. The only to improve accuracy would a chest CT scan.. I added this as a limitation of the study*

Table 4, line 268, what Wilcoxon test is indicated here?

***Answer:*** *.The Wilcoxon test for paired variables was used because the variables did not follow the normal distribution. When the table was added in the text, the caption that included the T-Test for paired variables was cut.*

Reviewer #2: The study by Silveira de Medeiros et al., performed in Brazil (a high tuberculosis endemic country), addressed the issue of false-negative tuberculin skin test (TST) results and interferon gamma release assays (IGRA) results in patients with psoriasis who are eligible for treatment with Methotrexate (MTX), and in those currently treated with MTX.

In the era of biological therapy for autoimmune diseases, adequate screening for latent TB infection (LTBI) before initiating treatment is key in preventing reactive TB. The authors justifiably address the difficulties in interpreting immunological test results (both TST and QuantiFERON Gold (QFT)) to detect an infection with Mycobacterium tuberculosis (mtb). In this study, both TST and QFT were performed in a BCG vaccinated cohort. Sociodemographic and clinical information as well as laboratory results were assessed. Varying TST cut-offs were used in order to evaluate the highest concordance between the tests before and after initiating treatment with MTX. The authors found that both negative and indeterminate QFT results changed randomly, regardless of treatment with MTX. Both TST induration and IFN-ý increased significantly after treatment and agreement nor comparison between TST and IGRA differed significantly before or after treatment.

The authors found some interesting results although the number of patients was very low. As this is not the first study evaluating TST/IGRA results in patients treated with MTX, I would suggest that the authors conduct a power analysis and subsequently include more patients. Secondly, the authors may evaluate QFT results quantitatively instead of binary (and subsequently recalculate agreement).

Major comments:
- No power analysis was described. This would have been in particular relevant since a large number of patients was excluded (n=20/45). Furthermore, what proportion of patients was expected to have a positive TST/QFT result?

***Answer:*** *I described the power of the analysis on the sample calculation on the text. I added a comparison of the frequency of LTBI with other studies. The LTBI average is 25%, with 5% conversions with induced booster effect. ( TST repeated after only one month after the first) . My average was similar but with 20% conversions with the repeated test after 12 weeks.*

*Patients were excluded because many had laboratory changes that would prevent the use of MTX. This is almost a real-life study. The profile of patients in the service is composed of people over the age of 40, with many comorbidities and in general hepatitis due to fatty liver desease. As there was a follow-up period to check whether patients would develop TB, and also was observed the loss of response to MTX, I had to be strict in the inclusion criteria. Otherwise many would have their medication suspended after 3 months.*

- In only 13 patients at least one positive TST or IGRA result was seen. Despite discovering significant differences, this number was too low to draw any conclusions.

***Answer:***

*The largest study is retrospective, with 70 patients, but 90% with negative results (60) for both tests ^1-6^. My study have more positive results proportionally than the others.*

*Larger studies are simpler, do not compare tests, do not mesure does not measure cytokines because they are very expensive, do not have follow-up, or include group of patients with different diseases, which is a way to inflate the N of the research, ^7^ being that this is actually a selection bias.*

1. Martyn-Simmons CL, Mee JB, Kirkham BW, Groves RW, Milburn HJ. Evaluating the use of the interferon-gamma response to *Mycobacterium tuberculosis*-specific antigens in patients with psoriasis prior to antitumour necrosis factor-alpha therapy: a prospective head-to-head cross-sectional study. Br J Dermatol. 2013;168: 1012-1018.
2. Latorre I, Mínguez S, Carrascosa JM, et al. Immune-mediated inflammatory diseases differently affect IGRAs' accuracy for latent tuberculosis infection diagnosis in clinical practice. *PLoS One*. 2017;12(12):e0189202. Published 2017 Dec 7. doi:10.1371/journal.pone.0189202.
3. de Andrade Lima E, de Andrade Lima M, de Lorena VM, de Miranda Gomes Y, Lupi O, Benard G, et al. Evaluation of an IFN-gamma assay in the diagnosis of latent tuberculosis in patients with psoriasis in a highly endemic setting. Acta Derm Venereol. 2011;91: 694-697.
4. Tavast E, Tuuminen T, Pakkanen SH, Eriksson M, Kantele A, Jarvinen A, et al. Immunosuppression adversely affects TST but not IGRAs in patients with psoriasis or inflammatory musculoskeletal diseases. Int J Rheumatol. 2012;2012: 381929.
5. Martyn-Simmons CL, Mee JB, Kirkham BW, Groves RW, Milburn HJ. Evaluating the use of the interferon-gamma response to *Mycobacterium tuberculosis*-specific antigens in patients with psoriasis prior to antitumour necrosis factor-alpha therapy: a prospective head-to-head cross-sectional study. Br J Dermatol. 2013;168: 1012-1018.
6. Sauzullo I, Mengoni F, Marocco R, Potenza C, Skroza N, Tieghi T, et al. Interferon-gamma release assay for tuberculosis in patients with psoriasis treated with tumour necrosis factor antagonists: *in vivo* and *in vitro* analysis. Br J Dermatol. 2013;169: 1133-1140.
7. Ramos JM, Masia M, Rodriguez JC, Lopez C, Padilla S, Robledano C, et al. Negative effect of immunosuppressive therapy in the performance of the QuantiFERON gold in-tube test in patients with immune-mediated inflammatory diseases. Clin Exp Med. 2013;13: 177-186.

- IGRA was interpreted in two and three categories (negative, positive; negative, positive, indeterminate resp.). QFT results were evaluated binary. In recent literature regarding, borderline QFT results (ranging from 0.15/0.20 to 0.70) are used as well, since a majority of borderline results reflects an infection with mtb (2017 Nemes Am resp j. Crit care med; 2017 Uzorka ERJ). Can the authors re-analyze their data using negative, borderline negative/positive, and positive (<0.15; 0.15-0.35/0.70; above 0.70 IU/mL) in order to improve agreement between TST and IGRA?

***Answer:*** *We do not have access to the quantiferon interferon levels. Only to the final result released by the analysis system. Therefore the only way to meet this fact was to consider the indeterminetes as positives. The analysis of the agreement between the tests was not an objective in itself, it was used to verify if there was a difference between the agreements before and after the treatment with MTX.*

- While the aim was to investigate whether or not MTX affected (agreement between) TST and IGRA results, corresponding clinical relevance, as stated in the introduction, was not sufficiently addressed. Please do so.

***Answer:*** *Thanks for the criticism. I modified the discussion trying to address this topic better.*

Minor comments:
- Line 178: what was the rationale for evaluating IFN-y response in pg/mL instead of IU/mL, as is customary in literature?

***Answer****: The manufacturers' recommendations were strictly followed and this was the parameter provided by him.*

- What was the rationale of reclassifying the indeterminate IGRA results as positive?

***Answer*** *IGRA showed agreement within the same range with two or three variables, so there is no difference in analyzing the results in two or three categories. Other authors also support that the indeterminate results are mostly due to positive results. ^2^ However, for the association with continuous variables, the groups would be very small.*

1. [*Uzorka JW*](https://www.ncbi.nlm.nih.gov/pubmed/?term=Uzorka%20JW%5BAuthor%5D&cauthor=true&cauthor_uid=29146607)*,* [*Kroft LJM*](https://www.ncbi.nlm.nih.gov/pubmed/?term=Kroft%20LJM%5BAuthor%5D&cauthor=true&cauthor_uid=29146607)*,* [*Bakker JA*](https://www.ncbi.nlm.nih.gov/pubmed/?term=Bakker%20JA%5BAuthor%5D&cauthor=true&cauthor_uid=29146607)*,* [*van Zwet EW*](https://www.ncbi.nlm.nih.gov/pubmed/?term=van%20Zwet%20EW%5BAuthor%5D&cauthor=true&cauthor_uid=29146607)*,* [*Huisman E*](https://www.ncbi.nlm.nih.gov/pubmed/?term=Huisman%20E%5BAuthor%5D&cauthor=true&cauthor_uid=29146607)*, [Knetsch-Prins C](https://www.ncbi.nlm.nih.gov/pubmed/?term=Knetsch-Prins%20C%5BAuthor%5D&cauthor=true&cauthor_uid=29146607),* [*van der Zwan CJ*](https://www.ncbi.nlm.nih.gov/pubmed/?term=van%20der%20Zwan%20CJ%5BAuthor%5D&cauthor=true&cauthor_uid=29146607)*,  [Ottenhoff THM](https://www.ncbi.nlm.nih.gov/pubmed/?term=Ottenhoff%20THM%5BAuthor%5D&cauthor=true&cauthor_uid=29146607),* [*Arend SM*](https://www.ncbi.nlm.nih.gov/pubmed/?term=Arend%20SM%5BAuthor%5D&cauthor=true&cauthor_uid=29146607)*. Proof of concept that most borderline Quantiferon results are true antigen-Specific responses.* [*Eur Respir J.*](https://www.ncbi.nlm.nih.gov/pubmed/?term=Proof+of+concept+that+most+borderline+Quantiferon+results+are+true+antigen-specific+responses) *2017 Nov 16;50(5).*

- What was the relevance of including Figure 1 and 2? Can both be omitted? ***Answer***: *The figures were removed*

- Line 114 TBLI not defined, probably LTBI. ***Corrected***

- Line 150 horas, please correct. ***Corrected***

- Line 163 (Cellestis, Melbourne, Australia) can be omitted as it was stated before. ***Corrected***

- Line 238 no units for IFN-y values are described, please correct. ***Corrected***

- Line 262-263 “This-markers”. Interpretation of such results are preferably described in the discussion part ***Corrected***

- Line 114 TBLI not defined, probably LTBI. ***Corrected***

- Line 303 “Studies – results”. Both TST and IGRA results are affected by immunosuppression, however, it differs substantially among the type of immunosuppression used, please illustrate.

***Corrected***

- Please correct the use of English grammar, for instance a consistent use of the past tense throughout the article is lacking, please check and correct. ***Corrected***
